# Comprehensive clinicopathological significance and putative transcriptional mechanisms of Forkhead box M1 factor in hepatocellular carcinoma

**DOI:** 10.1186/s12957-023-03250-z

**Published:** 2023-11-25

**Authors:** Hua-Yu Wu, Li-Feng Luo, Fang Wei, Hong-Mian Jiang

**Affiliations:** 1 Department of Medical Experimental Center, The First People’s Hospital of Nanning, Nanning, 530021 Guangxi Zhuang Autonomous Region People’s Republic of China; 2 Department of Pathology, The First People’s Hospital of Nanning, Nanning, 530021 Guangxi Zhuang Autonomous Region People’s Republic of China

**Keywords:** Forkhead box M1, Hepatocellular carcinoma, Tissue microarray, Immunohistochemistry, High throughput sequencing, Transcriptional regulation

## Abstract

**Background:**

The Forkhead box M1 factor (FOXM1) is a crucial activator for cancer cell proliferation. While FOXM1 has been shown to promote hepatocellular carcinoma (HCC) progression, its transcriptional mechanisms remain incompletely understood.

**Methods:**

We performed an in-house tissue microarray on 313 HCC and 37 non-HCC tissue samples, followed by immunohistochemical staining. Gene chips and high throughput sequencing data were used to assess FOXM1 expression and prognosis. To identify candidate targets of FOXM1, we comprehensively reanalyzed 41 chromatin immunoprecipitation followed by sequencing (ChIP-seq) data sets. We predicted FOXM1 transcriptional targets in HCC by intersecting candidate FOXM1 targets with HCC overexpressed genes and FOXM1 correlation genes. Enrichment analysis was employed to address the potential mechanisms of FOXM1 underlying HCC. Finally, single-cell RNA sequencing analysis was performed to confirm the transcriptional activity of FOXM1 on its predicted targets.

**Results:**

This study, based on 4235 HCC tissue samples and 3461 non-HCC tissue samples, confirmed the upregulation of FOXM1 in HCC at mRNA and protein levels (standardized mean difference = 1.70 [1.42, 1.98]), making it the largest multi-centered study to do so. Among HCC patients, FOXM1 was increased in Asian and advanced subgroups, and high expression of FOXM1 had a strong ability to differentiate HCC tissue from non-HCC tissue (area under the curve = 0.94, sensitivity = 88.72%, specificity = 87.24%). FOXM1 was also shown to be an independent exposure risk factor for HCC, with a pooled hazard ratio of 2.00 [1.77, 2.26]. The predicted transcriptional targets of FOXM1 in HCC were predominantly enriched in nuclear division, chromosomal region, and catalytic activity acting on DNA. A gene cluster encoding nine transcriptional factors was predicted to be positively regulated by FOXM1, promoting the cell cycle signaling pathway in HCC. Finally, the transcriptional activity of FOXM1 and its targets was supported by single-cell analysis of HCC cells.

**Conclusions:**

This study not only confirmed the upregulation of FOXM1 in HCC but also identified it as an independent risk factor. Moreover, our findings enriched our understanding of the complex transcriptional mechanisms underlying HCC pathogenesis, with FOXM1 potentially promoting HCC progression by activating other transcription factors within the cell cycle pathway.

**Supplementary Information:**

The online version contains supplementary material available at 10.1186/s12957-023-03250-z.

## Background

Liver cancer is a leading cause of cancer-related deaths. According to the latest data [1], primary liver cancer accounted for 41,210 new cases and 29,380 deaths, with a 5-year survival rate of only 21%, which is one of the lowest among all malignant tumors. Hepatocellular carcinoma (HCC) is the most common histological type, representing 85% of cases [2], and its complex etiology remains poorly understood. Treatment options include surgical resection, liver transplantation, and frequency ablation, but these are usually ineffective for late-stage diagnoses, which are common due to the difficulty in early detection. Recurrence rates after surgery can be as high as 21% [3]. Given these challenges, identifying new biomarkers for early diagnosis, targeted therapy, and prognosis has become a top priority.

As a transcription factor, Forkhead box M1 (FOXM1) is part of the Forkhead family and located at chromosome 12p13.3. It regulates gene expression during G2 phase, mediates G2/M progression, and maintains chromosome stability and mitosis normalcy [4, 5]. FOXM1 is upregulated in several cancers including lung [6], renal cell [7], ovarian [8], breast [9], and colorectal cancers [10]. In HCC [11], FOXM1 expression is elevated and has been linked to activation of Nrf2, inhibition of E-cadherin, targeting of CCNB1 and PBK, as well as generation, migration, proliferation, and poor prognosis [12]. FOXM1 may also be involved in sorafenib resistance [13]. Despite certain findings, the transcriptional mechanisms behind FOXM1’s role in HCC remain unclear and require further elucidation.

To this end, the present study aimed to confirm the clinicopathological significance of FOXM1 and investigate its transcriptional activity in relation to HCC progression.

## Methods

### Expression status of FOXM1 in HCC

#### Expression of FOXM1 in HCC based on the immunohistochemistry

A total of four tissue microarrays, including LVC1504, LVC2281, LVC481, and LVC482, were purchased from Pantomics, Inc. The sample inclusion standards were as follows: (I) tissue samples were pathologically diagnosed with primary HCC and (II) HCC and non-HCC tissue were sufficient for conducting immunohistochemical (IHC) experiments. To validate the protein expression of FOXM1 in HCC tissue, formalin-fixed and paraffin-embedded HCC and noncancerous samples in different tissue microarrays underwent a dehydration process. This was followed by inhibition of endogenous peroxidase activity and antigen retrieval. IHC staining was performed using a two-step method. For the IHC staining, a recombinant anti-FOXM1 antibody (ab207298) from Abcam Co., Ltd. [EPR17379] was purchased. In the experimental group, the anti-FOXM1 antibody was used as the primary antibody and phosphate-buffered saline served as a control. Both were incubated overnight at 37 °C. Subsequently, a secondary antibody was added to the sample and incubated for half an hour at 25 °C. Ten representative regions from each section with the strongest immunoreactivity were examined at 400 × magnification to assess immunostaining. FOXM1 expression was scored based on the number of positively expressed cells per 100 liver cancer or non-cancerous liver cells, with the signal located in the nucleus exhibiting varying intensities of yellow, brown, or dark brown. The relative protein expression level of FOXM1 in the specimens was determined by calculating the average value of positive cells across ten fields. All the experimental procedures were approved by the Ethics Committee of Pantomics, Inc.

#### Expression of FOXM1 in HCC based on gene chips and high-throughput sequencing data

HCC gene chips and high-throughput sequencing data were downloaded from the Gene Expression Omnibus (GEO) database, sequence read archive, ArrayExpress, and The Cancer Genome Atlas-The Genotype Tissue Expression (TCGA-GTEx) database, using the search keywords (Forkhead box M1 OR FOXM1 OR FOXM1A OR FOXM1B OR FOXM1C) AND (hepatocellular OR liver OR hepatic OR HCC). Two independent researchers conducted the search, with discrepancies resolved by consensus. GEO datasets were merged if they belonged to the same platform, followed by batch effect removal with the SVA package in R (version 4.2.1). We extracted FOXM1 expression in HCC and non-HCC groups. Scatter plots and receiver operating characteristic (ROC) curves were drawn using ggplot2 (version 3.4.1) and pROC (version 1.17.0.1) to compare FOXM1 expression between HCC tissues and controls, with AUC of ROC curves indicating the potential diagnostic marker for HCC when AUC ≥ 0.7.

#### Combined expression level of FOXM1 in HCC tissue

The expression of FOXM1 in HCC was analyzed through pooled data from in-house IHC, TCGA-GTEx, and GEO databases to calculate the standardized mean difference (SMD). The heterogeneity was measured by *I*^*2*^, where *I*^*2*^ > 50% indicated strong heterogeneity and a random-effects model was used. Publication bias was assessed by Egger’s test, Begg’s test, and funnel plot dissymmetry test, where *p* < 0.05 showed significant publication bias. A summary ROC (sROC) curve was drawn to show FOXM1 discrimination ability in HCC. Finally, pooled sensitivity and specificity were calculated using the meta package (version 4.18–2).

#### Correlation between the clinical features and FOXM1 expression in HCC

The clinical HCC samples provided different clinical parameters, and two investigators collected additional clinical features including gender, age, race, TNM stage, and American Joint Committee on Cancer (AJCC) pathologic stage in a blind manner. FOXM1 expressions were then compared across various clinicopathological characteristics to identify differences in FOXM1 expression levels among different subgroups of HCC.

### The prognosis of FOXM1 in HCC

We extracted overall survival (OS) information and status from both the in-house HCC cohort and TCGA-HCC cohort. HCC patients were defined as the FOXM1 high expression group or FOXM1 low expression group using the surv_cutpoint function in survminer (version 0.4.9) package. Survival analysis was conducted and visualized using survival (version 3.3–1) and survminer (version 0.4.9) packages to assess FOXM1 prognosis in HCC. Prognosis data for FOXM1 in HCC were also obtained from previous reports using the search strategies above, and high-quality articles were assessed using the Newcastle–Ottawa Scale with a maximum score of 9. A score ≥ 7 was deemed high quality, and hazard ratios (HR) were pooled.

### Potential mechanism of FOXM1 in HCC

#### Candidate targets of FOXM1 based on the Cistrome DB database

To investigate the candidate target genes of FOXM1 in various tissue samples, we utilized the Cistrome DB database to identify regulated sites of FOXM1. We downloaded a total of 41 datasets and selected candidate targets with a score ≥ 1 [14].

#### Relative genes of FOXM1 in HCC based on high-throughput datasets

The relative genes of FOXM1 in HCC were identified using gene chips and high-throughput sequencing datasets. Co-expressed genes (CEGs) were filtered based on Pearson’s correlation analysis of the TCGA database and merged GEO datasets, selecting genes with a correlation coefficient ≥ 0.30 and *p* < 0.05. The final set of relative genes comprised genes that co-expressed with FOXM1 in no less than 20 platforms.

#### Highly expressed genes of HCC based on high-throughput datasets

To identify highly expressed genes (HEGs) in HCC, SMD values were calculated from gene chips and high-throughput datasets [15]. HEGs were defined as those with an SMD > 0 between cancerous and non-tumorous samples, with a statistical significance of *p* < 0.05.

#### Potential biological function of FOXM1 in HCC

The potential targets of FOXM1 in HCC were identified by taking an intersection of candidate targets from Cistrome DB, CEGs, and HEGs from gene chips and high-throughput datasets. Then the intersection genes combined with FOXM1 were put into the analysis of the potential biological function of FOXM1 in HCC. The analysis was based on Gene Ontology (GO) annotation and Kyoto Encyclopedia of Genes and Genomes (KEGG) enrichment analysis by using clusterProfiler (version 4.7.1.003). Then, a protein–protein interaction (PPI) network was constructed by the Search Tool for the Retrieval of Interacting Genes (STRING) database, and it was visualized by Cytoscape (version 3.9.0).

#### Verification of target genes for FOXM1 in HCC

The regulatory relationship between FOXM1 and the target gene was verified using chromatin immunoprecipitation sequencing (ChIP-seq) technology to show the intensity and position of the peak, with the application of the Cistrome DB. Furthermore, the correlation between FOXM1 expression and its targets was determined by using bulk RNA sequencing and single-cell RNA sequencing analysis [16].

### Statistical analysis

We employed R software to perform the Wilcox test and the Kruskal–Wallis test while analyzing diverse clinicopathological characteristics associated with FOXM1. In order to determine statistical significance for HR at a 95% confidence interval (CI), it must not include “1” within the CI.

## Results

### Upregulation of FOXM1 in HCC tissues

#### Expression of FOXM1 in HCC based on immunohistochemistry

**Fig. 1:**
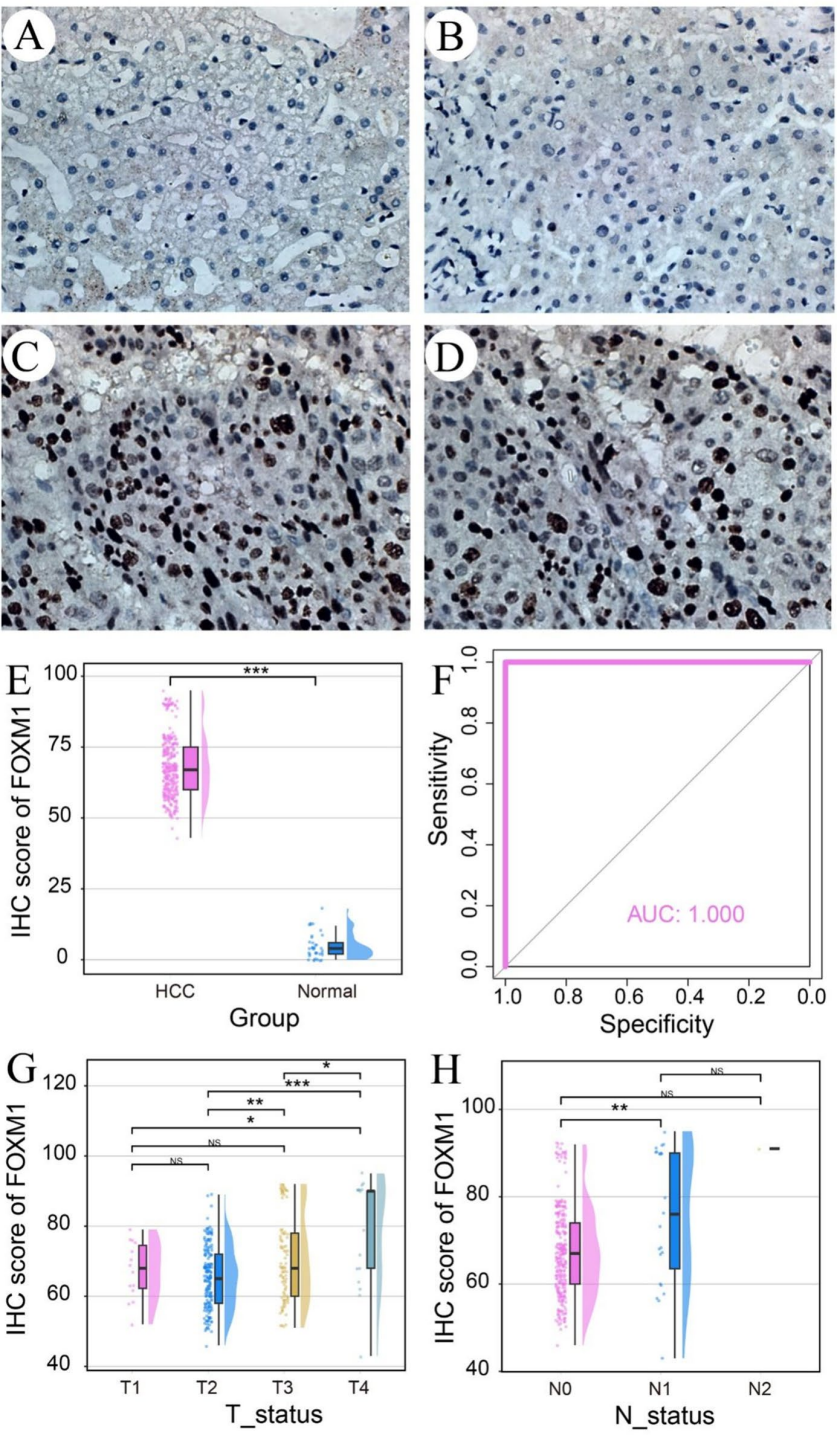
FOXM1 expression in non-HCC and HCC samples based on in-house immunohistochemistry. The expression status and clinicopathological significance of FOXM1 protein were explored in non-HCC samples (**A**, **B**) and HCC samples (**C**, **D**). **E** FOXM1 protein expression in the HCC group was obviously elevated. **F** Increased expression of FOXM1 protein could be used to discriminate HCC from non-HCC tissue. **G**, **H** Increased expression of FOXM1 protein could be found in HCC tissue with advanced tumor status and lymphatic metastasis. *p < 0.05; **p < 0.01; ***p < 0.001; NS, not significant

A total of 313 HCC tissue and 37 non-HCC tissue were ultimately included for IHC staining. The clinical features of the included 313 HCC patients were shown in Supplemental Table 1. Compared to non-HCC samples (Fig. 1A, B), HCC samples showed significantly higher FOXM1 expression and a larger number of FOXM1-positive cells (Fig. 1C, D). The upregulation of FOXM1 in HCC samples was further confirmed by a Wilcox test (*p* < 0.0001, Fig. 1E). With an AUC value approaching 1, high expression of FOXM1 protein was identified as a promising potential biomarker for discriminating HCC (Fig. 1F). Notably, FOXM1 protein expression increased with advanced tumor status (Fig. 1G) and lymphatic metastasis (Fig. 1H) in HCC tissues.

#### Expression of FOXM1 in HCC based on gene chips and high-throughput databases

**Fig. 2:**
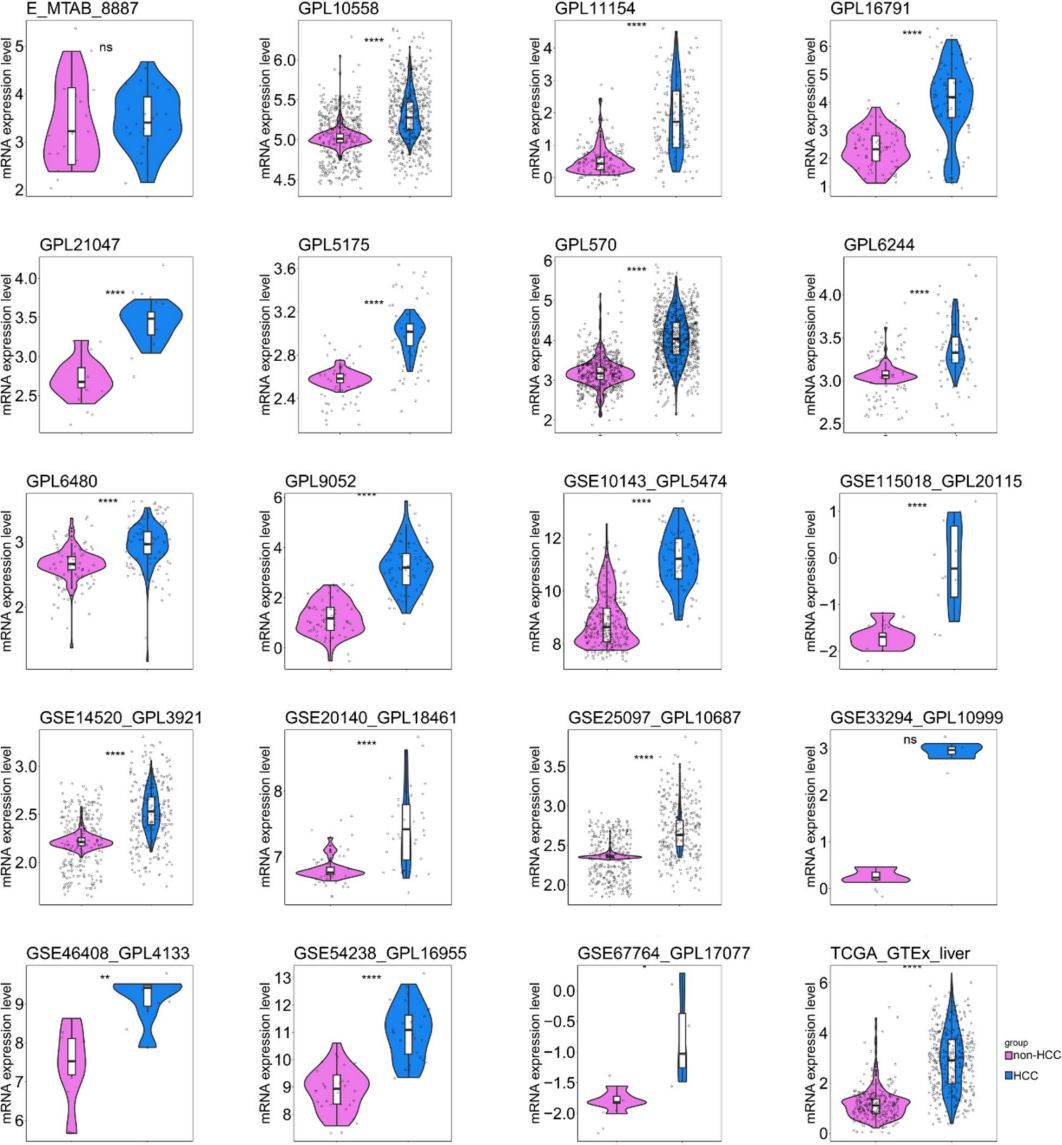
The expression status of FOXM1 mRNA in HCC and non-HCC tissue samples. *p < 0.05; **p < 0.01; ***p < 0.001; ****p < 0.0001; NS, not significant

**Fig. 3:**
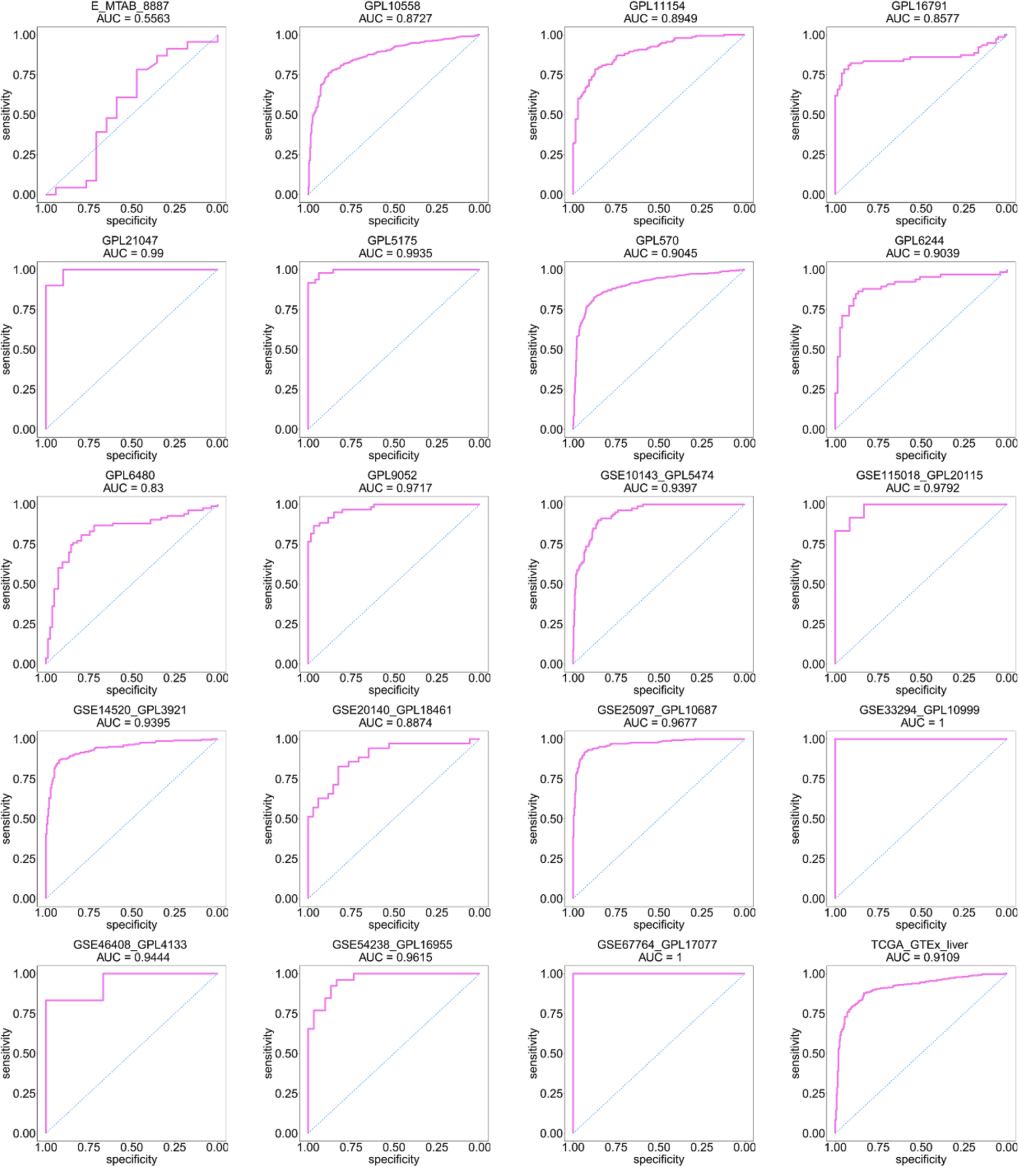
The discrimination ability of FOXM1 mRNA in HCC and non-HCC tissue samples

We obtained 83 datasets from the aforementioned databases, which we combined into 39 platforms. Figures 2 and 3 and Supplemental Figs. 1 and 2 present scatter plots and ROC curves depicting the expressions of FOXM1 across each platform. Our results demonstrated that FOXM1 was upregulated in HCC tissue and possessed significant discriminatory power.

#### Combined expression level of FOXM1 in HCC tissues

**Fig. 4:**
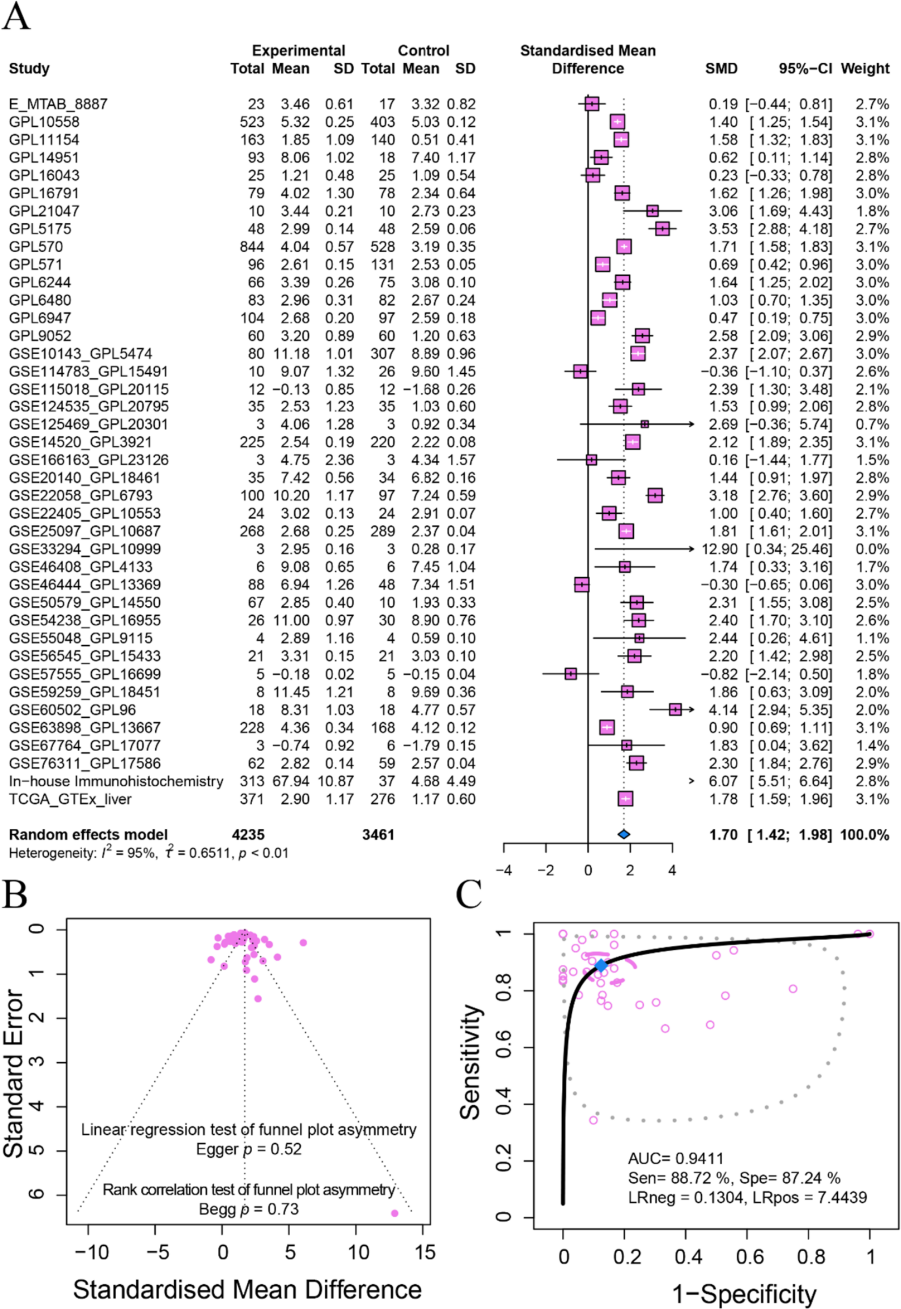
Comprehensive expression status of FOXM1 in HCC tissue. The expression level of FOXM1 was explored by integrating the in-house immunohistochemistry, gene chips, and high-throughput sequencing data. **A** Forest plots. SMD = 1.70, 95%CI 1.42–1.98. **B** Funnel diagram with Begg’s test and Egger’s test. The test results indicated no significant bias between the included datasets. **C** sROC curve. The AUC value of the sROC curve further confirmed that FOXM1 had a strong discrimination ability in HCC (AUC = 0.94)

**Fig. 5:**
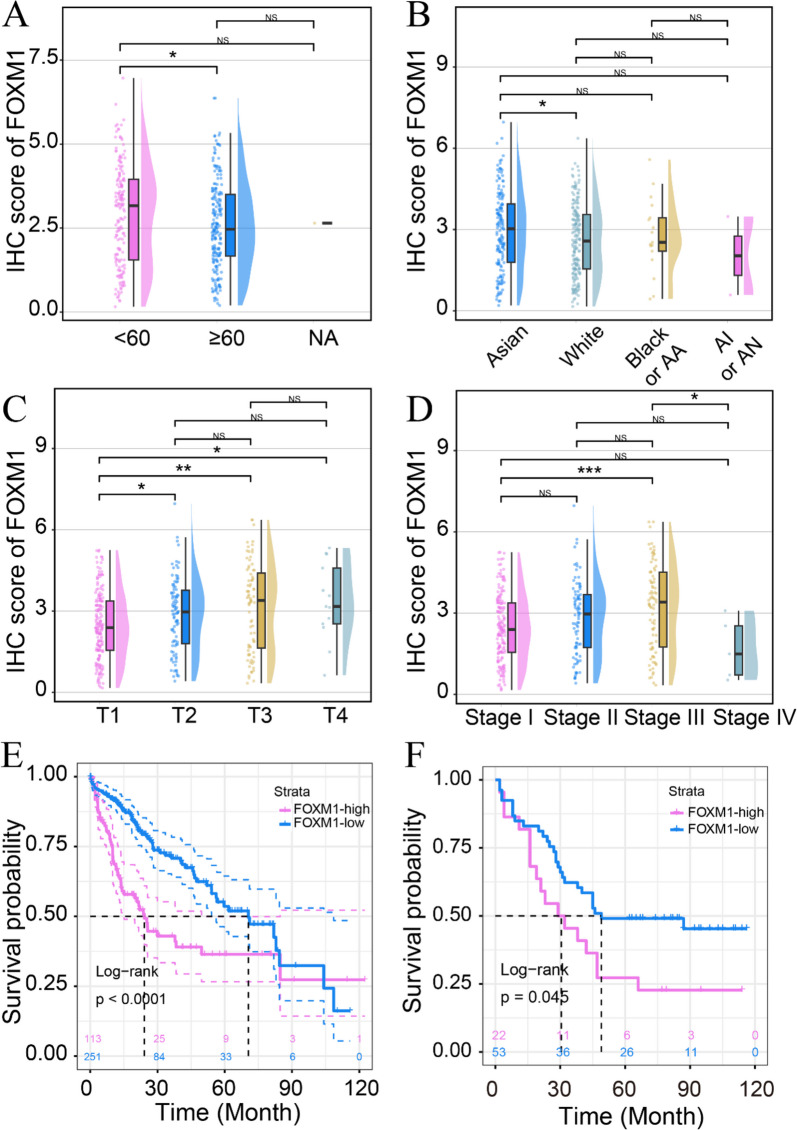
FOXM1 expression in HCC subgroups based on high throughput sequencing analysis. The expression of FOXM1 mRNA was significantly higher in HCC patients with younger ages and poorer tumor status. **A** Age. **B** Race. **C** Tumor status. **D** American Joint Committee on Cancer pathologic stage. HCC patients were grouped into two groups by using the surv_cutpoint function in survminer (version 0.4.9). The prognostic value of FOXM1 was confirmed by using TCGA (**E**) and in-house immunohistochemistry (**F**). AA, African American; AI, American Indian; AN, Alaska native. *p < 0.05; **p < 0.01; ***p < 0.001; NS, not significant

To verify the expression status, we calculated and pooled SMD values of FOXM1 from in-house IHC, gene chips, and high-throughput sequencing data sets. The studies involved showed great heterogeneity (*I*^*2*^ = 95%, *p* < 0.01), so we used the random-effects model. The pooled SMD was 1.70 (95% CI 1.42–1.98; HCC, 4235 samples; non-HCC, 3461 samples), indicating upregulation of FOXM1 in HCC. The funnel diagram showed no publication bias in the inclusion studies (Begg’s *p* = 0.73, Egger’s *p* = 0.52, Fig. 4B). The sROC curve confirmed that FOXM1 had strong discriminatory significance in HCC (AUC = 0.94, sensitivity = 88.72%, specificity = 87.42%, negative likelihood ratio = 0.13, positive likelihood ratio = 7.44) (Fig. 4C and Supplemental Fig. 3). Additionally, FOXM1 expression was closely related to age, race, tumor status, and AJCC pathologic stage (Fig. 5A–D). Higher protein expression FOXM1 was found in HCC patients with younger age (≥ 60 *v.s.* < 60) and poorer tumor status (T2 *v.s.* T3, T1 *v.s.* T2, and Stage I *v.s.* Stage III).

### The clinical prognosis value of FOXM1 upregulation in HCC

Based on data from the in-house HCC cohort and TCGA cohort, high expression of FOXM1 is associated with poor prognosis in HCC patients (Fig. 5E, F). Moreover, univariable and multivariable Cox regression analysis showed that FOXM1 mRNA may be an independent risk factor for HCC (Table 1). Upon pooling overall survival data from the in-house HCC cohort and 14 other studies using a fixed-effects model, increased FOXM1 expression was confirmed as a risk factor for HCC patients, potentially affecting their prognosis (pooled HR value = 2.00, 95%CI 1.77–2.26, Supplemental Fig. 4A). No publication bias was detected from the funnel plot (Supplemental Fig. 4B), and the Galbraith plot indicated that the pooled HR result was stable.

**Table 1:** Univariable and multivariable Cox regression analysis of FOXM1 in hepatocellular carcinoma

		All	HR (univariable)	HR (multivariable)
FOXM1	Mean ± SD	2.7 ± 1.4	1.30 (1.15–1.47, *p* < 0.001)	1.27 (1.12–1.45, *p* < 0.001)
Age	Mean ± SD	59.6 ± 13.4	1.01 (1.00–1.03, *p* = 0.073)	/
Sex	Female	119 (32.7%)	/	/
	Male	245 (67.3%)	0.82 (0.57–1.16, *p* = 0.258)	/
AJCC pathologic stage	I	169 (46.4%)	/	/
	II	84 (23.1%)	1.43 (0.87–2.33, *p* = 0.155)	1.30 (0.78–2.15,* p* = 0.313)
	III	83 (22.8%)	2.71 (1.78–4.13, *p* < 0.001)	2.48 (1.60–3.85, *p* < 0.001)
	IV	4 (1.1%)	5.72 (1.76–18.56, *p* = 0.004)	0.00 (0.00–Inf, *p* = 0.997)
	V	24 (6.6%)	2.83 (1.54–5.21, *p* < 0.001)	1.72 (0.86–3.44, *p* = 0.126)
*T* status	T1	179 (49.2%)	/	/
	T2	91 (25.0%)	/	/
	T3	78 (21.4%)	/	/
	T4	13 (3.6%)	/	/
	Tx	1 (0.3%)	/	/
	Na	2 (0.5%)	/	/
*N* status	N0	247 (67.9%)	/	/
	N1	4 (1.1%)	2.13 (0.52–8.71, *p* = 0.292)	1.17 (0.28–4.87, *p* = 0.830)
	Nx	112 (30.8%)	1.50 (1.03–2.17,* p* = 0.032)	1.34 (0.76–2.34, *p* = 0.309)
	Na	1 (0.3%)	2.33 (0.32–16.83, *p* = 0.401)	1.42 (0.18–10.84, *p* = 0.738)
M_status	M0	262 (72.0%)		
	M1	3 (0.8%)	4.28 (1.34–13.60, *p* = 0.014)	242,816.50 (0.00–Inf, *p* = 0.996)
	Mx	99 (27.2%)	1.61 (1.11–2.33, *p* = 0.013)	1.43 (0.83–2.49, *p* = 0.200)
Race	White	182 (50.0%)	/	/
	Asian	155 (42.6%)	/	/
	Black or African American	16 (4.4%)	/	/
	American Indian or Alaska Native	1 (0.3%)	/	/
	Na	10 (2.7%)	/	/
Ethnicity	Not Hispanic or Latino	329 (90.4%)	/	/
	Hispanic or Latino	17 (4.7%)	0.91 (0.40–2.06, *p* = 0.814)	/
	Na	18 (4.9%)	1.28 (0.62–2.62, *p* = 0.506)	/

### Potential molecular mechanism of FOXM1 in HCC

**Fig. 6:**
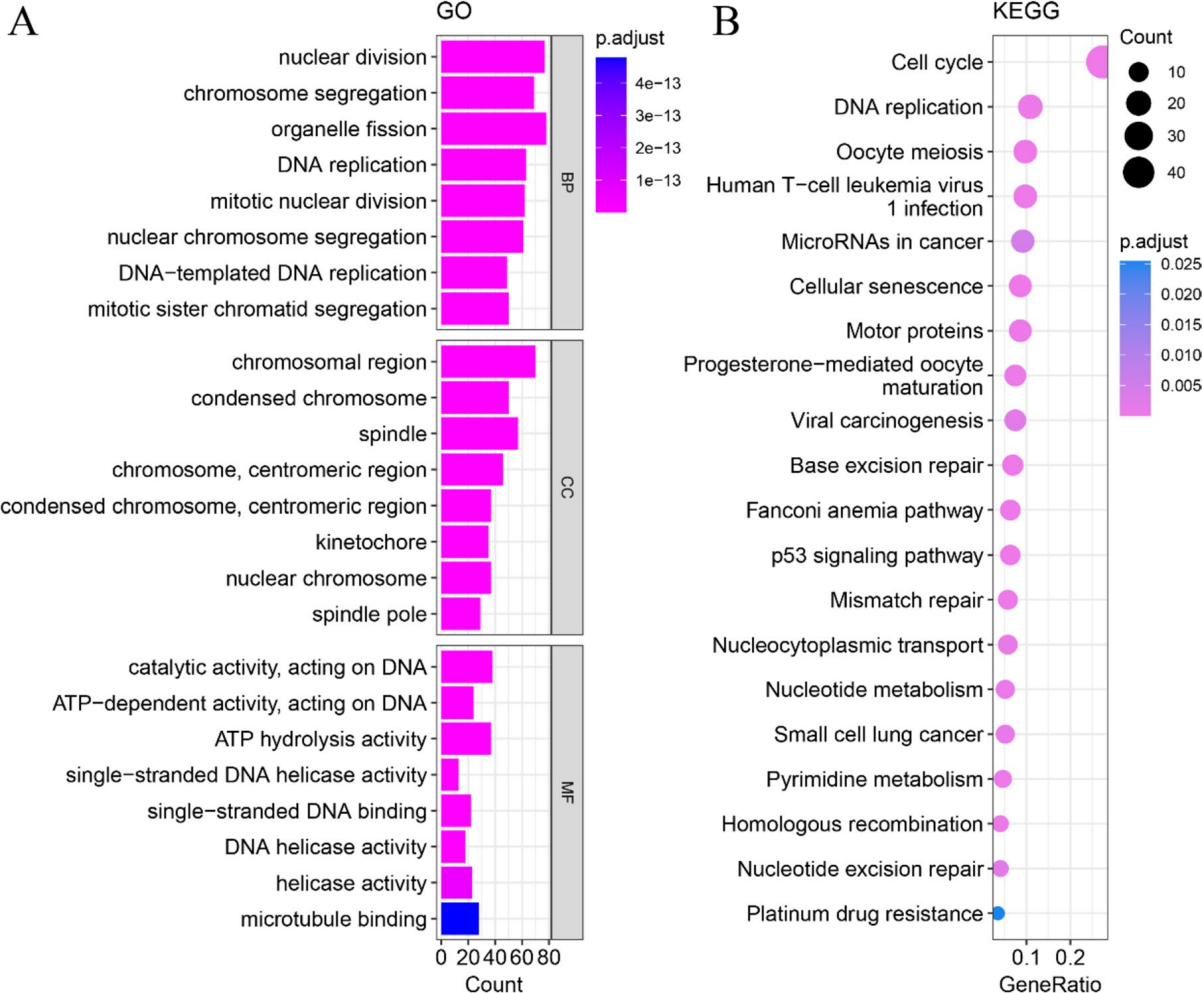
GO and KEGG enrichment analysis based on the predicted transcriptional targets of FOXM1 factor. The biological function of FOXM1 was explored by annotating the enriched GO and KEGG items of its targets. **A** Biological process, cellular compartment, and molecular function of GO analysis. **B** Result of KEGG analysis. The color of the dot represents the adjusted *p* value of each enriched item and the size of the dot reflects the genes enriched to the corresponding items

**Fig. 7:**
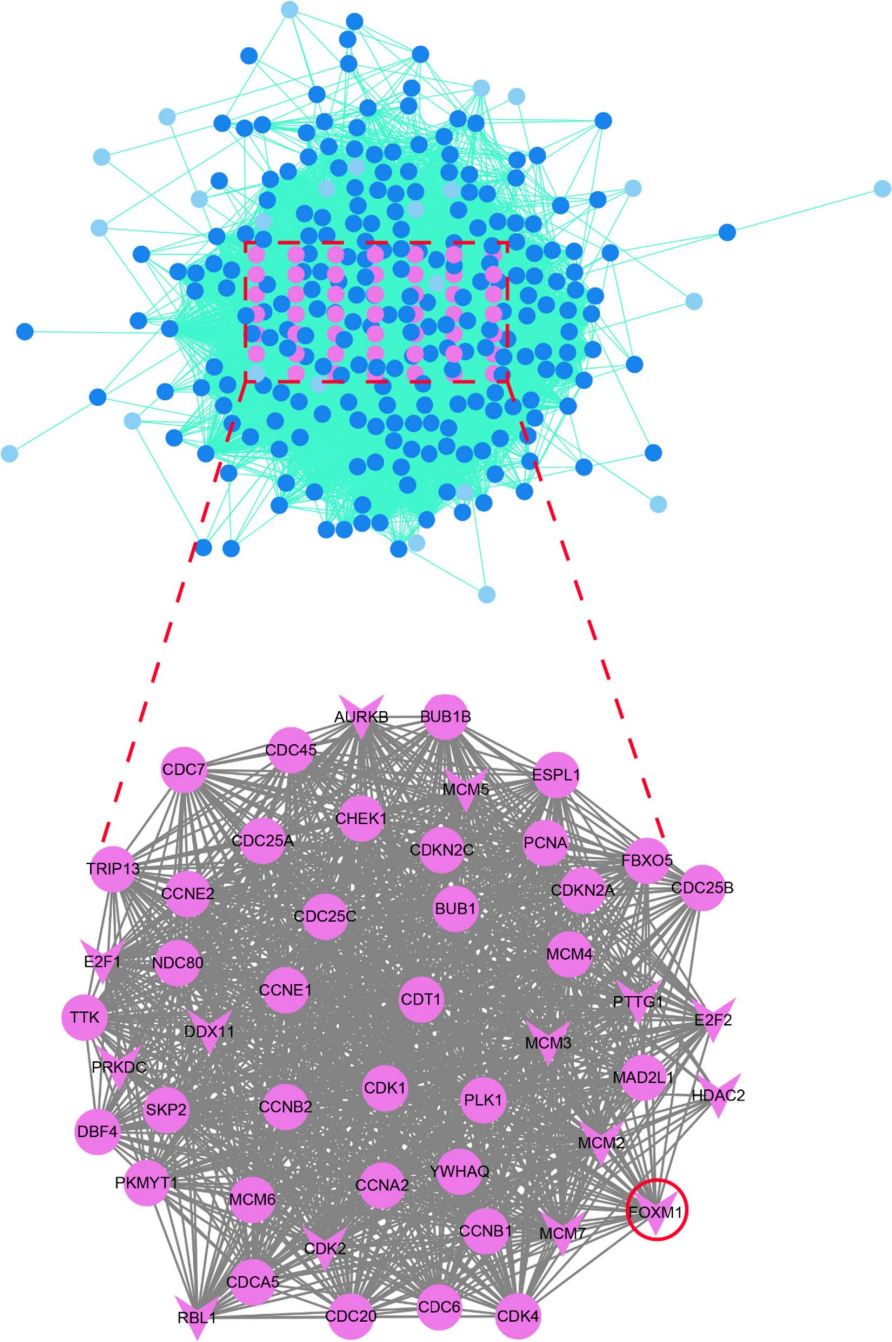
PPI network analysis of FOXM1 factor and its predicted targets in HCC. The predicted target genes of FOXM1 were significantly aggregated in the cell cycle pathway and were colored in pink. The V-shaped symbol represented the transcription factor, while the circle represented the encoded protein

In HCC, a total of 313 intersection genes were predicted as potential targets of FOXM1. GO analysis indicated that the target genes enriched in biological processes such as nuclear division, chromosome segregation, and organelle fission; in cellular components such as chromosomal region, condensed chromosome, and spindle; and in molecular functions such as catalytic activity acting on DNA, ATP-dependent activity acting on DNA, and ATP hydrolysis activity (Fig. 6A). KEGG analysis revealed that the intersection genes were mainly involved in the cell cycle, DNA replication, mismatch repair, and base excision repair (Fig. 6B). A PPI network based on the intersection genes was constructed (Fig. 7), predicting a gene cluster encoding nine transcriptional factors (AURKB, E2F1, HDAC2, MCM2, MCM3, MCM5, MCM7, PTTG1, and RBL1) that were positively regulated by FOXM1, promoting the cell cycle signaling pathway in HCC.

### Verification of target genes for FOXM1 in HCC

**Fig. 8:**
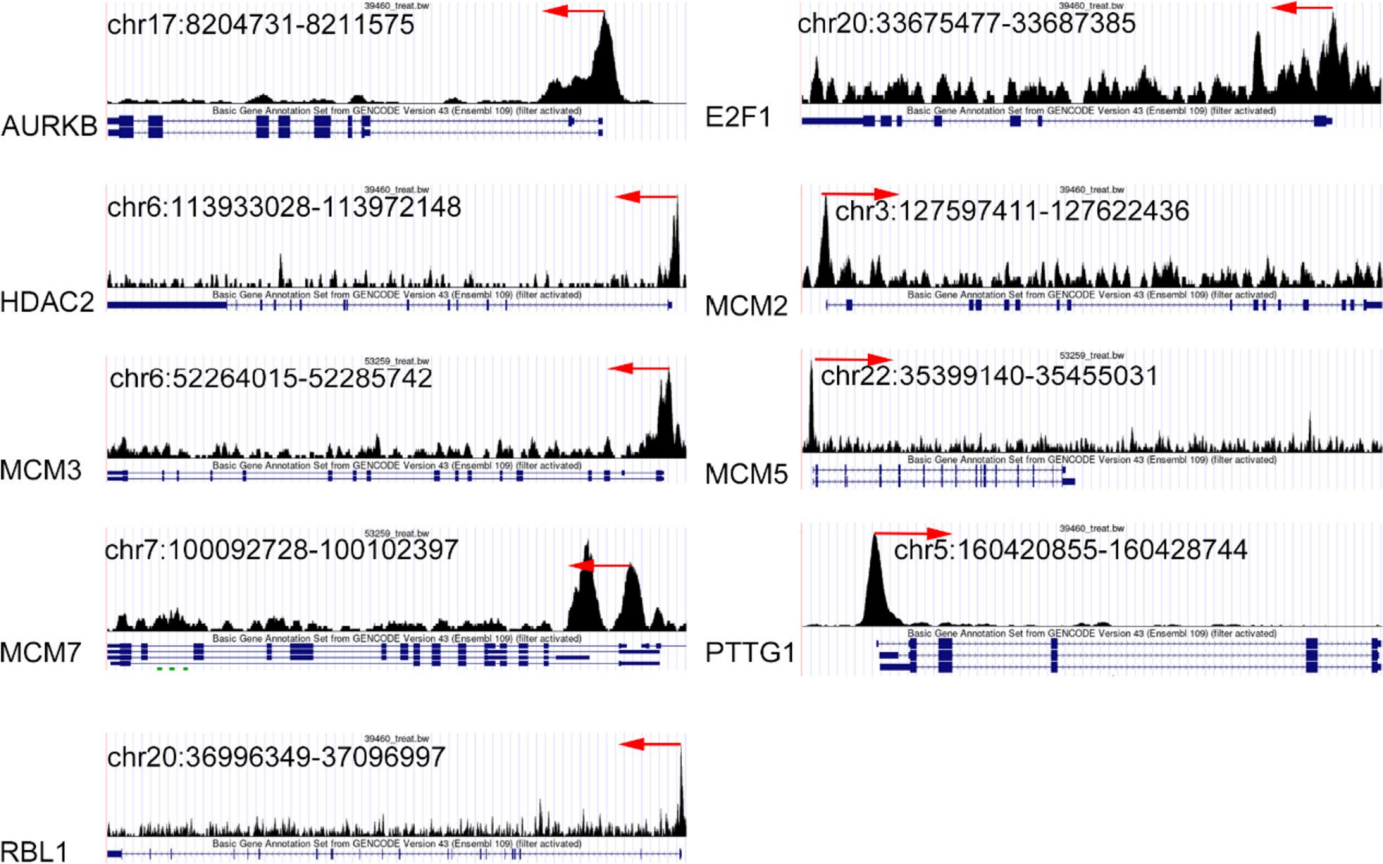
Prospective transcriptional activity of FOXM1 factor in the cell cycle pathway of HCC. The position and the intensity of the peak indicated that FOXM1 might bind at the upstream of its targets. The red arrow indicates the direction of transcription for genes

**Fig. 9:**
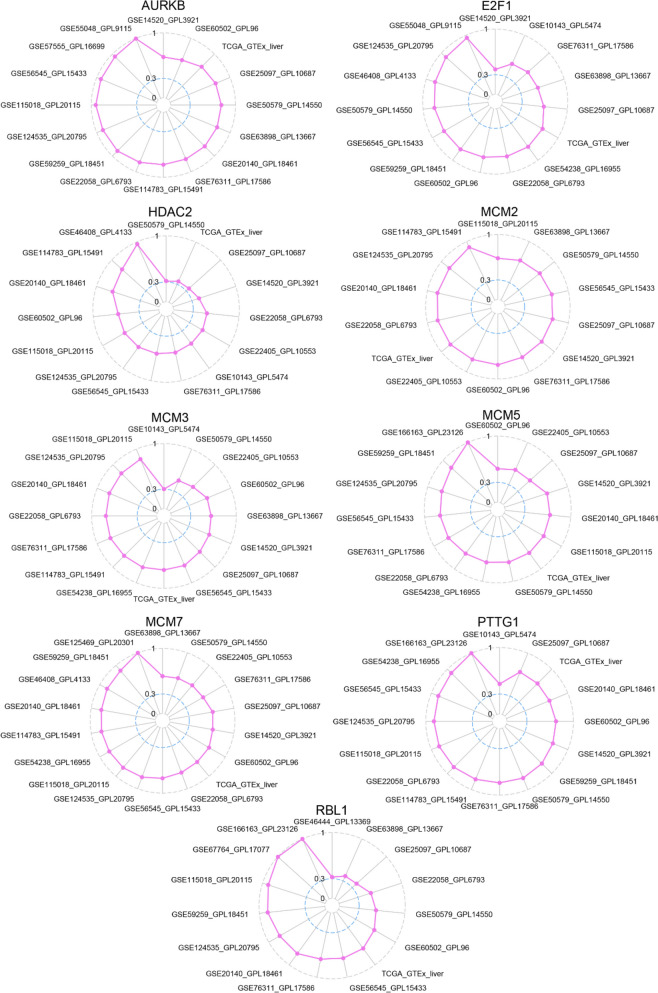
Co-expression between FOXM1 factor and its prospective transcriptional targets in the cell cycle pathway of HCC

**Fig. 10:**
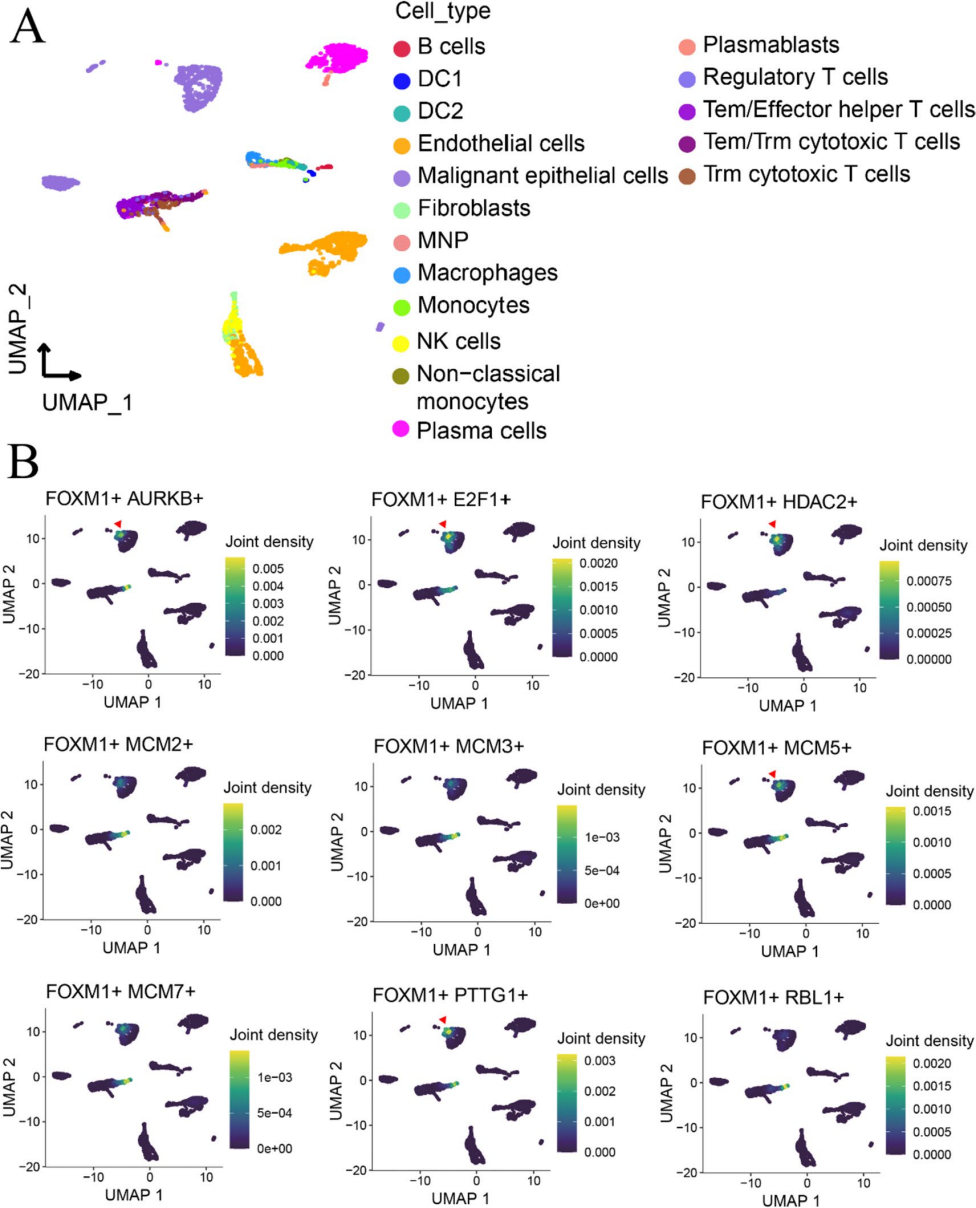
Single-cell transcriptome analysis of FOXM1 factor and its prospective transcriptional targets in the cell cycle pathway of HCC. The expression intensity and distribution were explored in HCC single cells by using GSE125449. **A** Dimension reduction of HCC single cells by using uniform manifold approximation and projection. **B** As was marked by red arrow, FOXM1 was co-expressed with its putative transcriptional targets in malignant epithelial cells

Based on the Cistrome DB database, the position and intensity of the peak indicated that FOXM1 may bind to the upstream regions of nine target genes (Fig. 8). These nine target genes were significantly upregulated in HCC tissues (SMD ranging from 0.64 to 1.99, *p* < 0.05) and showed a positive correlation with FOXM1 expression (Fig. 9; Pearson correlation coefficient ≥ 0.30, *p* < 0.05). Additionally, we successfully detected malignant epithelial cells from HCC single cells (see Fig. 10A) and demonstrated the co-expression of FOXM1 with its potential transcriptional targets, particularly AURKB, E2F1, HDAC2, MCM5, and PTTG1 (Fig. 10B). This finding provides preliminary evidence supporting the transcriptional activity of FOXM1 and its associated targets.

## Discussion

The present study on FOXM1 in HCC stands out from previous research due to its comprehensive analysis of expression patterns and clinical implications of FOXM1 mRNA and protein using a multi-centered approach. The study integrated tissue microarray, high throughput sequencing data sets, gene microarrays, and literature-based results to provide insights into potential molecular mechanisms underlying the onset and progression of HCC. More importantly, our investigation explored the transcriptional targets of FOXM1, offering feasible therapeutic targets for patients with late-term HCC.

Our study confirmed the upregulation of FOXM1 in HCC and established its prognostic value using 4235 HCC tissues and 3461 non-HCC tissues from 13 countries, including Canada, China, France, Germany, India, Italy, Japan, Singapore, South Korea, Spain, Switzerland, Turkey, and the USA. FOXM1 expression was consistently elevated at both mRNA and protein levels, supporting previous studies [17–20]. Notably, FOXM1B and FOXM1C isoforms, which are transcriptional activators, were responsible for FOXM1 upregulation in cancer according to a recent study [21]. Our research also demonstrated a significant association between high FOXM1 levels and poor prognosis for HCC patients. The pooled results of HR indicated that FOXM1 was an independent risk factor and a promising prognostic biomarker for HCC patients, consistent with earlier studies [22–24]. Furthermore, we observed elevated protein expression of FOXM1 in Asian individuals, as well as in patients with advanced-stage HCC. Notably, HCC patients under the age of 60 exhibited higher levels of FOXM1 expression. Thus, upregulated FOXM1 may be a potential biomarker in HCC and is correlated with unfavorable prognostic conditions.

Furthermore, this study provided important insights into the biological function of FOXM1. In this study, a highly connected transcriptional regulatory network of FOXM1 was established by integrating HCC HEGs, FOXM1 CEGs, and ChIP-seq-identified candidate targets relevant to cell cycle processes such as nuclear division, chromosome segregation, organelle fission, chromosomal region, spindle, DNA replication, mismatch repair, and base excision repair. FOXM1 is a key transcription factor in cell cycle progression [19, 25–27], reaching peak expression during the S and G2/M phases [21], and promoting cell malignancy by influencing cell cycle, DNA repair, and control of cell division [28]. Although the exact transcriptional mechanisms by which FOXM1 participates in HCC formation are incompletely understood, the present study identified a gene cluster encoding nine transcription factors that may trigger cascades of the cell cycle pathway in HCC initiation and progression, with AURKB [29] and PTTG1 [30] confirmed as transcriptional targets of FOXM1 and E2F1 showing upregulation of FOXM1 protein expression in liver cancer cells in a nude mice model [31]. HDAC2, MCM2, MCM3, MCM5, MCM7, and RBL1 were also upregulated in HCC tissue and positively correlated to FOXM1 expression, with FOXM1 showing binding intensities upstream of their promoters, suggesting positive transcriptional regulation between them. These findings suggest that FOXM1 may be involved in HCC carcinogenesis and progression by activating the transcription of cell cycle regulatory factors and accelerating the cell cycle process, but further experimental evidence is necessary to confirm these hypotheses.

Finally, our study offers a new direction for research into anti-HCC drugs. FOXM1 has long been the target for developing anti-cancer agents [32]. According to a recent study [33], FOXM1 disruption may reverse sorafenib resistance of HCC cells; however, its efficiency in the clinical practice deserves validation. Herein, we predicted nine important transcriptional targets of FOXM1 by targeting the cell cycle pathway in HCC. Interestingly, AURKB was predicted as a target protein of Scutellaria barbata in treating HCC [34] and E2F1 inhibitor could be used to hamper the proliferation of HCC cells [35]. Additionally, HDAC2 [36], MCM7 [37], and PTTG1 [38] have also shown potential for treating cancers. Therefore, co-inhibition of FOXM1 and its transcriptional targets may be a potential therapeutic strategy for HCC treatment. Further studies are needed to verify this hypothesis.

The limitations of this study cannot be ignored. First, our study integrated data sets from various ethnicities, which provided a high degree of heterogeneity. To solve this issue, a randomized effect model was used in our statistical analysis. Second, the clinical implication of FOXM1 in HCC patients was based on in-house HCC samples and online databases. However, the practical applications of FOXM1 in the prognosis of HCC patients remain unclear. Therefore, further clinical practice is required to examine the actual value of FOXM1 in HCC. Third, the molecular mechanism of FOXM1 depends on in silico analysis. In vitro and in vivo experiments are required to confirm the roles of FOXM1 in HCC and its potential therapeutic targets.

## Conclusions

This study not only confirmed the upregulation of FOXM1 in HCC but also identified it as an independent risk factor. Moreover, our findings enriched our understanding of the complex transcriptional mechanisms underlying HCC pathogenesis, with FOXM1 potentially promoting HCC progression by activating other transcription factors within the cell cycle pathway.

### Supplementary Information

**Additional file 1: Figure S1.** The scatter plots showed the expression status of FOXM1 mRNA in HCC and non-HCC tissue samples. * *p*<0.05; **p<0.01; ***p<0.001;****p<0.0001; NS,not significant. **Figure S2. **The ROC curves showed the discrimination ability of FOXM1 mRNA in HCC and non-HCC tissue samples. **Figure S3. **Accuracy of FOXM1 overexpression in discriminating HCC and non-HCC tissue. (A) Forest plot of sensitivity (B) Forest plot of specificity (C) Forest plot of positive likelihood ratio (D) Forest plot of negative likelihood ratio. **Figure S4. **The comprehensive prognostic significance of FOXM1 in HCC based on the in-house immunohistochemistry, scientific literature, and high throughput sequencing data. (A) Forest plot of hazard ratio (B) Funnel plot with Begg’s test and Egger’s test (C) Galbraith plot.

**Additional file 2: Table S1.**

## Data Availability

The data that support the findings of this study are available from the Gene Expression Omnibus (GEO) database (http://www.ncbi.nlm.nih.gov/geo), the ArrayExpress (http://www.ebi.ac.uk/arrayexpress/), the sequence read archive (SRA) (http://ncbiinsights.ncbi.nlm.nih.gov/tag/sra/), and TCGA-GTEx databases.

## Abbreviations

FOXM1: Forkhead box M1 factor
HCC: Hepatocellular carcinoma
ChIP-seq: Chromatin immunoprecipitation followed by sequencing
IHC: Immunohistochemical
GEO: Gene Expression Omnibus
TCGA-GTEx: The Cancer Genome Atlas-The Genotype Tissue Expression
SMD: Standardized mean difference
sROC: Summary receiver operating characteristic
AJCC: American Joint Committee on Cancer
OS: Overall survival
HR: Hazard ratios
CEGs: Co-expressed genes
HEGs: Highly expressed genes
GO: Gene Ontology
KEGG: Kyoto Encyclopedia of Genes and Genomes
PPI: Protein–protein interaction
STRING: Search Tool for the Retrieval of Interacting Genes
CI: Confidence interval
