# Supplementary material for: Comprehensive clinicopathological significance and putative transcriptional mechanisms of Forkhead box M1 factor in hepatocellular carcinoma
Source: World J Surg Oncol. 2023 Nov 25;21:366. doi: 10.1186/s12957-023-03250-z (PMC10675979; doi:10.1186/s12957-023-03250-z)
Supplement: Supplementary file 1 — Additional file 1: Figure S1. The scatter plots showed the expression status of FOXM1 mRNA in HCC and non-HCC tissue samples. * p<0.05; **p<0.01; ***p<0.001;****p<0.0001; NS,not significant. Figure S2. The ROC curves showed the discrimination ability of FOXM1 mRNA in HCC and non-HCC tissue samples. Figure S3. Accuracy of FOXM1 overexpression in discriminating HCC and non-HCC tissue. (A) Forest plot of sensitivity (B) Forest plot of specificity (C) Forest plot of positive likelihood ratio (D) Forest plot of negative likelihood ratio. Figure S4. The comprehensive prognostic significance of FOXM1 in HCC based on the in-house immunohistochemistry, scientific literature, and high throughput sequencing data. (A) Forest plot of hazard ratio (B) Funnel plot with Begg’s test and Egger’s test (C) Galbraith plot. [file 12957_2023_3250_MOESM1_ESM.docx]

**Supplementary Materials**


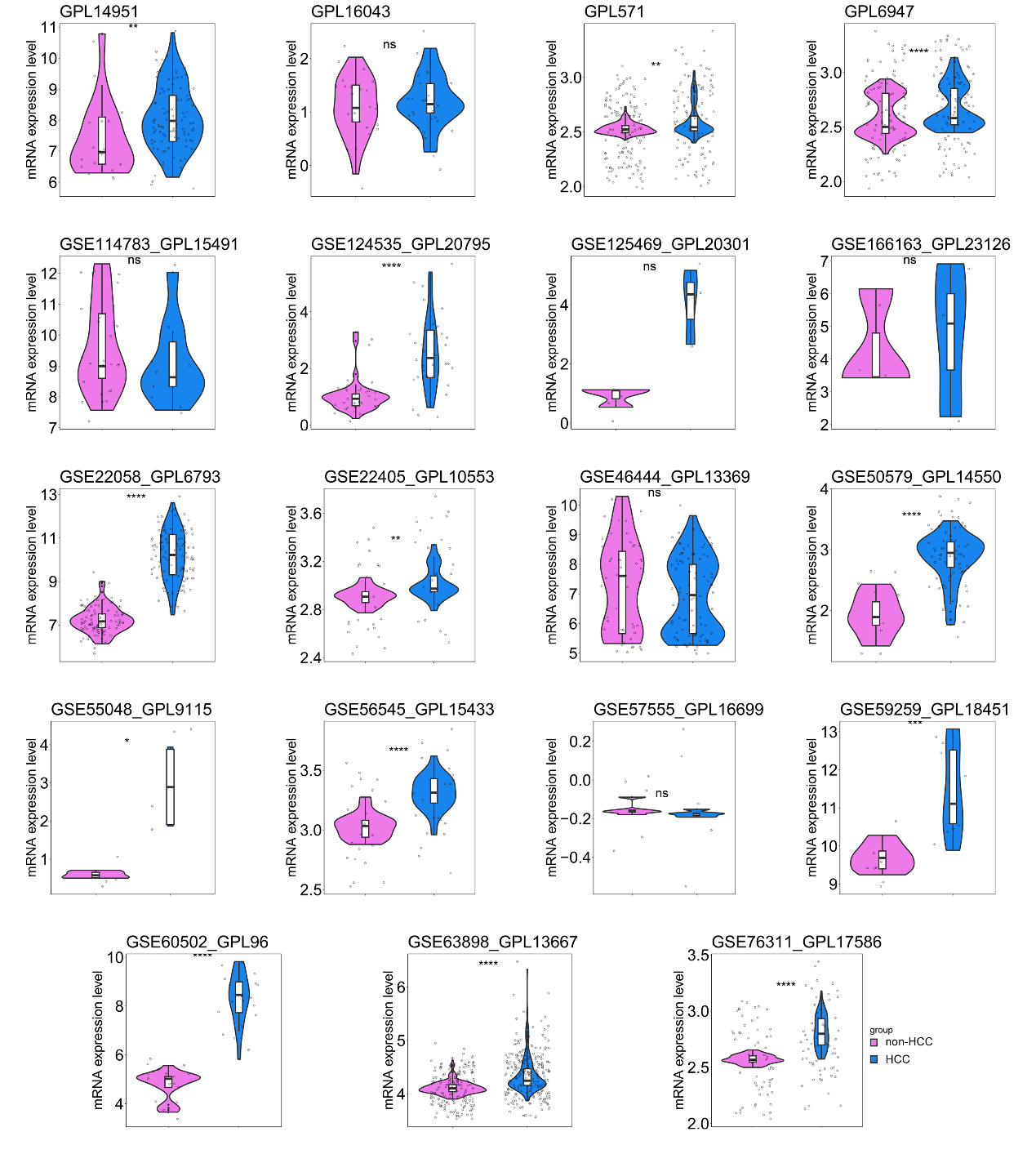


Supplemental Figure 1: The scatter plots showed the expression status of FOXM1 mRNA in HCC and non-HCC tissue samples.

* *p*<0.05; ** *p*<0.01; *** *p*<0.001; **** *p*<0.0001; NS, not significant


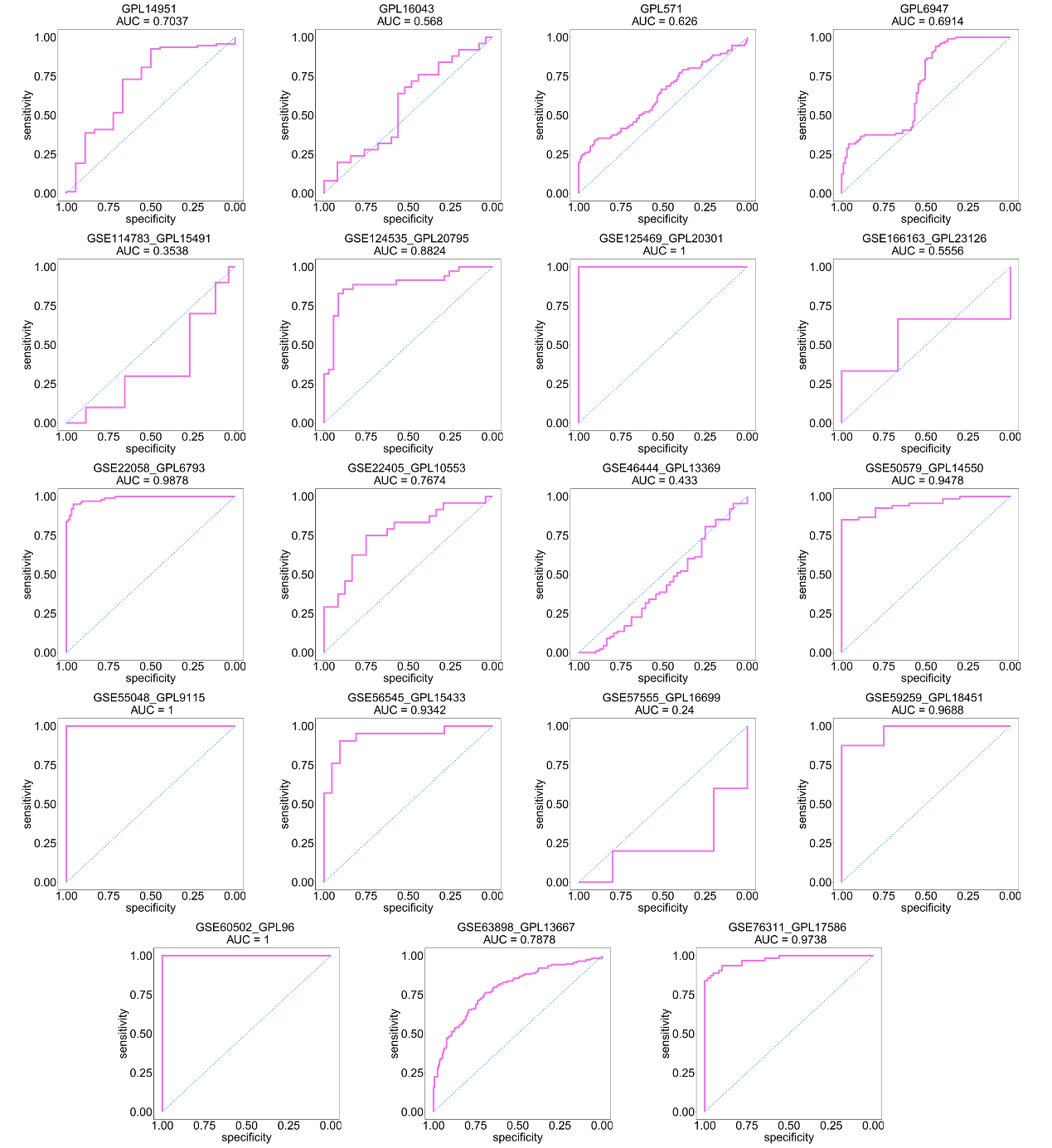


Supplemental Figure 2: The ROC curves showed the discrimination ability of FOXM1 mRNA in HCC and non-HCC tissue samples.


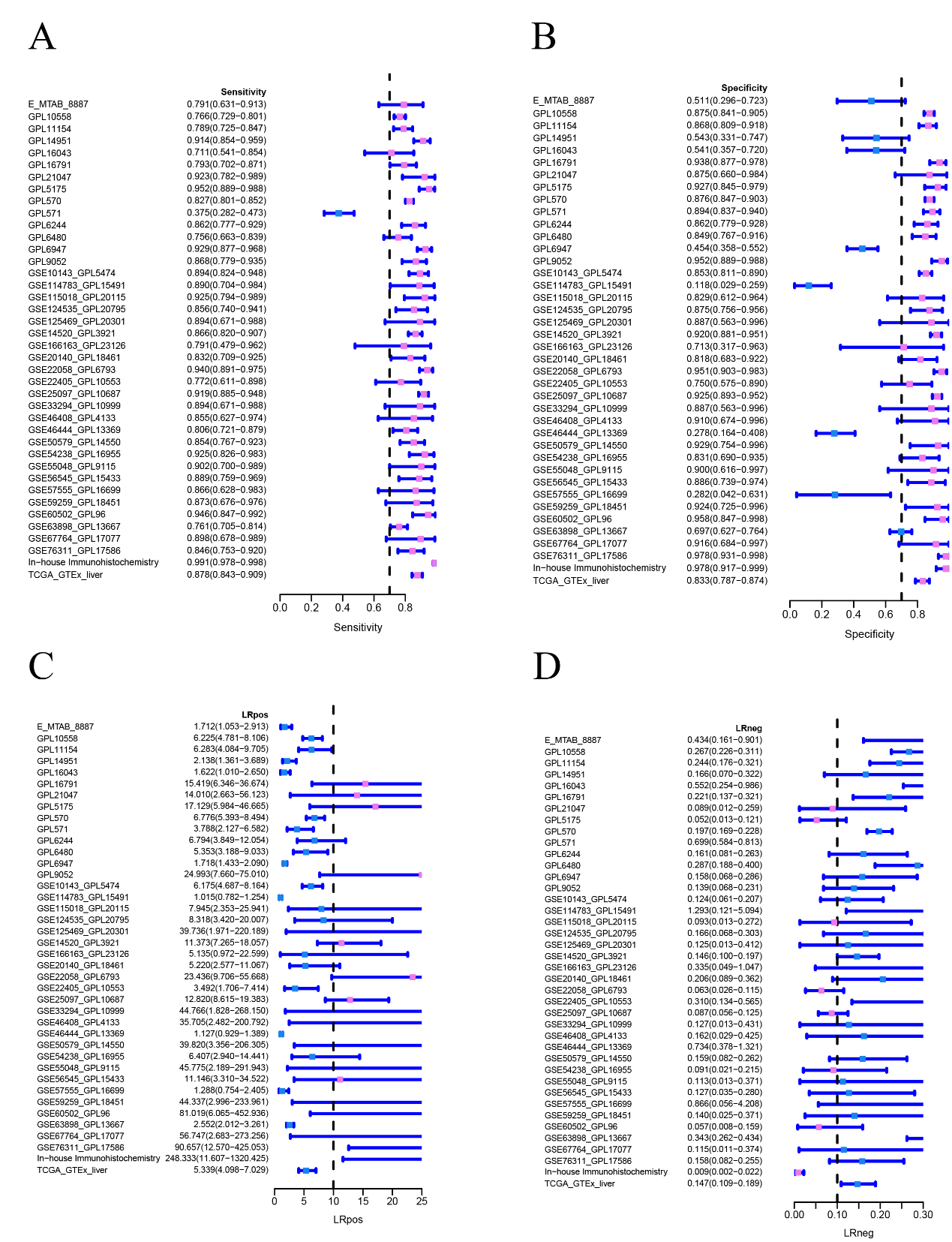


Supplemental Figure 3: Accuracy of FOXM1 overexpression in discriminating HCC and non-HCC tissue

(A) Forest plot of sensitivity (B) Forest plot of specificity (C) Forest plot of positive likelihood ratio (D) Forest plot of negative likelihood ratio


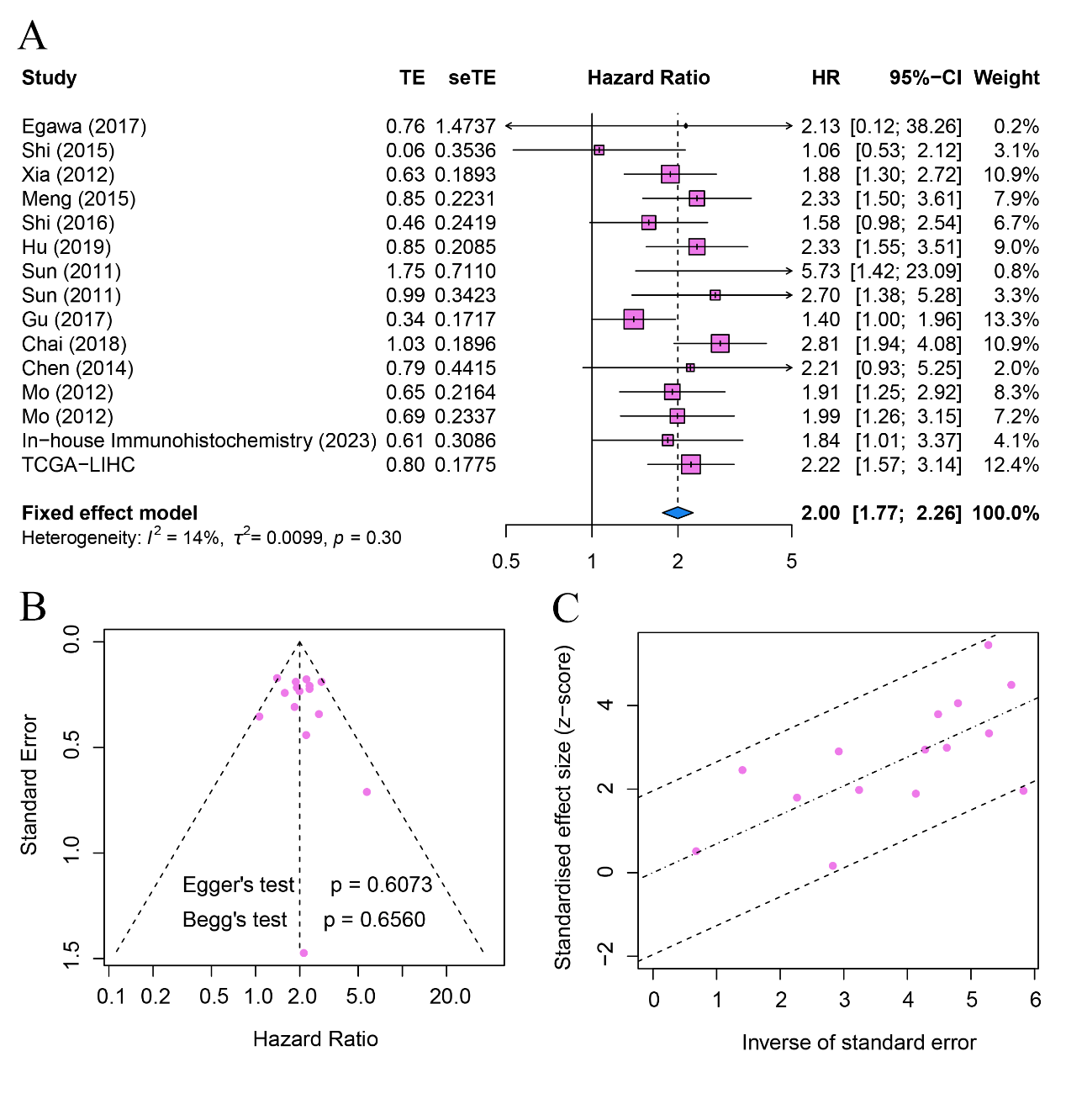


Supplemental Figure 4: The comprehensive prognostic significance of FOXM1 in HCC based on the in-house immunohistochemistry, scientific literature, and high throughput sequencing data.

(A) Forest plot of hazard ratio (B) Funnel plot with Begg’s test and Egger’s test (C) Galbraith plot.
